# Improved Blow-Pipe

**Published:** 1856-01

**Authors:** 


*Improved Blow-Pipe.*—Mr. Flemming, a student of the Baltimore College of Dental Surgery, recently presented to the editors, a model of a blowpipe, invented by himself, and by means of which, a continuous blast may be kept up. It is constructed with a bellows and a valve to prevent the air from returning when the mouth is taken from the mouth-piece, while the compression of the sides of the bellows by means of two springs, keeps up the current of air from the nozzle of the instrument, until a fresh supply is furnished by the mouth. We shall publish a brief description of the instrument furnished by the ingenious inventor in our next issue, together with a cut, showing the manner of its construction. We would have published it in this, if the diagram had been prepared in time.

